# Possible Vertical Transmission From an Unsuspected SARS-CoV-2-Infected Mother to Her Newborn

**DOI:** 10.7759/cureus.15717

**Published:** 2021-06-17

**Authors:** Ali Naseh, Sahar Ashrafzadeh

**Affiliations:** 1 Pediatrics, Mofid Children Hospital, Shahid Beheshti University of Medical Sciences, Tehran, IRN; 2 Pediatrics, Taleghani Hospital, Shahid Beheshti University of Medical Sciences, Tehran, IRN; 3 Pediatrics, David Geffen School of Medicine, University of California, Los Angeles, USA

**Keywords:** covid-19, sars-cov-2, newborn health, vertical infectious disease transmission, maternal-infant infection, pregnancy, infant, coronavirus infections, transplacental transmission, asymptomatic

## Abstract

Although the Coronavirus Disease 2019 (COVID-19) has been found to have multiple routes of transmission, limited data exist on whether the vertical transmission of severe acute respiratory syndrome coronavirus 2 (SARS-CoV-2) can occur from asymptomatic infected mothers to their newborns during pregnancy. We report a full-term newborn girl who was found to be positive for COVID-19 at 24 hours of life and subsequently symptomatic with fever, tachycardia, tachypnea, elevated lactate dehydrogenase, and elevated total bilirubin. The newborn was delivered by a mother who was not suspected of having COVID-19 before giving birth, but who developed fever and dyspnea five hours after delivery and was found to be positive for COVID-19. Upon further history collection, the mother reported recent mild nasal congestion in the days prior to delivery. This case highlights that the vertical transmission of COVID-19 to a newborn may occur late during the third trimester from a mother who was not suspected of having the infection. All pregnant women may need to be screened for COVID-19 symptoms, including non-specific symptoms, prior to admission for labor and delivery floors in order to perform diagnostic tests and recommended safety precautions to keep newborns and hospital personnel safe.

## Introduction

The recent outbreak of severe acute respiratory syndrome coronavirus 2 (SARS-CoV-2) has catalyzed an outburst of research on Coronavirus Disease 2019 (COVID-19). Although the virus has been found to have multiple routes of transmission, limited data exist on whether the vertical transmission of SARS-CoV-2 can occur from asymptomatic infected mothers to their newborns during pregnancy. Recent systematic reviews and case reports have not yet definitively verified the rate of vertical transmission during pregnancy [1-4]. We report a full-term newborn with positive SARS-CoV-2 reverse transcriptase-polymerase chain reaction (RT-PCR) 24 hours after delivery by an asymptomatic COVID-19 positive pregnant mother.

## Case presentation

A female neonate was delivered by cesarean section at 39 gestational weeks to a 39-year-old mother. The mother experienced gestational hypertension during the pregnancy, which she managed by diet and exercise, but otherwise, she did not have other medical conditions or take medications. The newborn weighed 3280 grams and had Apgar scores of nine and 10, at one and five minutes, respectively. The mother briefly breastfed the newborn once in the first three hours after birth, after which the newborn was removed from the mother’s room since the mother reported a sore throat.

**Figure 1:**
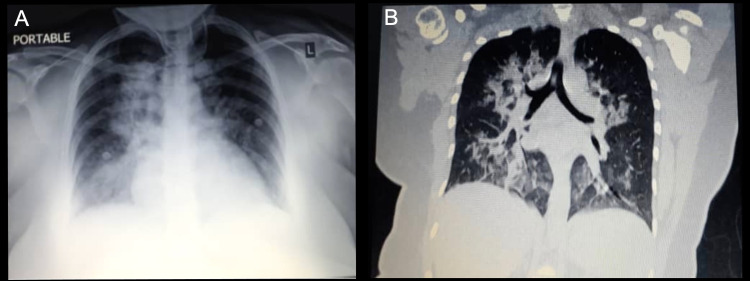
Imaging studies of the 39-year-old mother performed five hours after delivery when the mother developed acutely worsening dyspnea. (A) Chest radiograph and (B) computerized tomography scan revealed bilateral patchy airspace opacities.

Approximately five hours after delivery, the mother became febrile and began experiencing dyspnea. The mother was admitted to the intensive care unit, where she received supplemental oxygen through an oxygen mask. Her chest x-ray and computerized tomography scan revealed bilateral patchy airspace opacities (Figure 1). An RT-PCR of a nasopharyngeal swab was performed that was positive for SARS-CoV-2. Upon further questioning, the mother reported mild nasal congestion in the two weeks prior to delivery but otherwise denied any recent fever, cough, or other symptoms. She did not report the nasal congestion on the initial history prior to delivery because she did not suspect that she had COVID-19.

After the mother’s RT-PCR returned positive for SARS-CoV-2, the newborn was transferred to our hospital’s neonatal intensive care unit and placed in isolation in an incubator for concern of possible deterioration from SARS-CoV-2 infection. An RT-PCR was performed on the newborn’s nasopharyngeal swab at 24 hours of life that was positive for SARS-CoV-2. The newborn’s arterial blood gas was normal, and her oxygen saturation remained over 95% on the pulse oximeter. She remained afebrile, and her C-reactive protein was 5 mg/L (reference range: 0-10 mg/L), so it was decided that she would be monitored.

On day four, the newborn developed a fever of 39 degrees Celsius (102.2 degrees Fahrenheit), tachypnea (60 breaths/minute), tachycardia (170 beats/minute), and poor feeding. The newborn’s chest radiograph showed a non-significant haziness, and her chest CT scan was unremarkable. Her oxygen saturation remained above 94% on room air. A diagnostic sepsis workup was performed by obtaining blood, cerebrospinal fluid (CSF), and urine cultures. Her laboratory tests were notable for an elevated lactate dehydrogenase of 746 IU/L (reference range: 230-460 IU/L), elevated total bilirubin of 307.8 μmol/L (reference range: 3.42-17.1 μmol/L; or 18 mg/dL, reference range: 0.2-1.0 mg/dL), with direct bilirubin of 8.55 μmol/L (reference range: 1.71-5.13 μmol/L; or 0.5 mg/dL, reference range: 0.1-0.3 mg/dL). Otherwise, she did not have leukopenia, leukocytosis, lymphocytopenia, thrombocytopenia, or abnormal aspartate aminotransferase (AST) or alanine aminotransferase (ALT) levels. Her fever was managed by dabbing her with wet sponges, providing her with oral hydration, temporarily decreasing her incubator’s temperature, and empirically treating her with ampicillin and gentamycin as per protocol for febrile newborns [5]. She also underwent phototherapy, which reduced her total bilirubin to 136.8 μmol/L (8 mg/dL).

On day five, the newborn’s fever resolved, and she returned to feeding well and breathing with normal respiratory effort. An RT-PCR of her nasopharyngeal swab returned negative for SARS-CoV-2. On day eight, blood, urine, and CSF cultures remained negative, so antibiotics were discontinued, and the newborn was sent home in good health.

## Discussion

This case presents a newborn who tested positive for SARS-CoV-2 at 24 hours of life after being born to a SARS-CoV-2-positive mother who was nearly asymptomatic prior to and during the delivery. As no other hospital staff or patients at either hospital had COVID-19 on the delivery and labor floors, we believe that the virus was transmitted from the mother to the newborn through one of the following two transmission modalities.

The first transmission modality is intrauterine vertical transmission during pregnancy. The mother reported recent nasal congestion, which may have been due to COVID-19. The maternal viremia, even if transient, may have enabled transplacental transmission of the virus. Nearly 27%-45% of patients with SARS-CoV-2 have detectable viral RNA in their serum [6]. Furthermore, several other respiratory coronaviruses, such as 229E, OC43, NL63, and HKU1, have shown evidence of vertical transmission [7]. A few case reports of possible coronavirus transmission have revealed transient IgM antibodies in an otherwise asymptomatic newborn and a positive RT-PCR for SARS-CoV-2 in a pre-term newborn after the newborn had skin-to-skin contact on the sixth day of life with her mother, who was subsequently diagnosed with COVID-19 [3,8]. As the virus in our case was detected within the first day after delivery, and the estimated false-negative rate of RT-PCR for SARS-CoV-2 is nearly 100% on days one and two after exposure to SARS-CoV-2, it seems unlikely that the newborn would have tested positive within 24 hours after birth if she were exposed after delivery [9].

The second modality of possible SARS-CoV-2 transmission is horizontal transfer during the first three hours the newborn was exposed to her mother. The virus may have been transmitted through aerosolization of the mother’s respiratory droplets [10]. As the mother was neither sneezing nor coughing, which generate significantly more respiratory droplets compared to talking, and was wearing a mask as per hospital policy, the newborn’s exposure to SARS-CoV-2 was likely less than we typically expect from patients who sneeze or cough [11]. Alternatively, transmission through breast milk may have occurred since the newborn was breastfed once before being separated from her mother; however, this is unlikely since reports on the presence of SARS-CoV-2 RNA in breast milk are limited [12].

This case highlights that even asymptomatic pregnant women not suspected of having COVID-19 may transfer SARS-CoV-2 to their newborns either through vertical or horizontal transmission. The mother’s single symptom of mild nasal congestion during delivery highlights the need to establish screening guidelines for pregnant patients. One approach for clinicians to appropriately test and isolate patients may be to screen patients for COVID-19 symptoms, including mild and non-specific symptoms such as even subtle nasal congestion. Sutton et al. found that screening for COVID-19 symptoms led to identifying four (1.9%) of 215 pregnant women on the labor and delivery floor who tested positive for SARS-CoV-2 [13]. An alternative approach - universal testing of COVID-19 - can also be considered; recent studies implementing universal COVID-19 testing of pregnant women admitted to labor and delivery floors found that asymptomatic women tested positive for SARS-CoV-2 anywhere from 0.43% to 13.7%, suggesting that screening patients based only on symptoms and not diagnostic testing may lead to missing some patients [12,13]. A low (0.43%) SARS-CoV-2-positivity rate among asymptomatic patients was reported in an institution in California, with a lower COVID-19 community prevalence than that of New York City, where higher positivity rates (13.0% and 13.7%) were reported; therefore, institutions can make decisions on screening based on symptoms versus universal diagnostic testing by considering their community’s prevalence of COVID-19 [13,14].

In this case, the mother and newborn both had relatively mild COVID-19 disease outcomes. However, acute respiratory distress requiring intubation has been reported in pregnant women, who should be closely monitored after COVID-19 diagnoses [15]. Numerous studies have shown that newborns with COVID-19 have mild to no symptoms [16]; this newborn’s symptoms of fever, tachycardia, tachypnea, elevated lactate dehydrogenase (LDH), and elevated total bilirubin were transient and improved after one day. Unlike influenza, where maternal morbidity and mortality are higher than non-pregnant women, COVID-19 seems to be tolerated well among both newborns and many pregnant women [17].

The limitations of this study include not testing the amniotic fluid, breast milk, and placental blood for SARS-CoV-2 because the clinical team did not suspect that the mother had SARS-CoV-2 infection at the time of delivery. Although SARS-CoV-2 has rarely been identified in amniotic fluid or breast milk, a recent study found three (27%) of 11 PCR tests of placental and membrane swabs from women with COVID-19 to be positive, highlighting a possible route of vertical transmission [8,12,18].

## Conclusions

This case underscores the importance of actively screening pregnant women for COVID-19 infection even when they are asymptomatic and are not suspected to be infected. The newborns whose mothers are found to be COVID-19 positive should promptly get tested. We propose that vertical transmission of SARS-CoV-2 during the late third trimester of pregnancy may be possible as the newborn tested positive for the virus soon after delivery.
